# Influence of the family nucleus on obesity in children from northeastern Brazil: a cross-sectional study

**DOI:** 10.1186/1471-2458-7-235

**Published:** 2007-09-07

**Authors:** Ana M Oliveira, Antônio C Oliveira, Marcele S Almeida, Nelson Oliveira, Luis Adan

**Affiliations:** 1 Department of Pediatrics, Federal University of Bahia, School of Medicine Salvador, Bahia, Brazil Rua Padre Feijó, 240, 3° andar, Salvador, Bahia, Brazil. Zip Code 40110-170; 2 Department of Health, State University of Feira de Santana, Feira de Santana, Bahia, Brazil. Km 03 – Br 116 – Campus Universitário – Feira de Santana, Bahia, Brazil. Zip Code 40031-460; 3 Av. Maria Quitéria, 1660 – Feira de Santana-BA – Brazil, Zip Code 44025-260

## Abstract

**Background:**

Obesity is considered to be caused by a combination of heredity and environmental factors with typical onset during childhood. The aim of this study was to identify family risk factors for the development of obesity in children from Brazil.

**Methods:**

Cross-sectional study with 699 children, randomly and proportionally selected, ranging from 5 to 9 years of age, from public and private schools in Feira de Santana-BA. Overweight and obesity were defined using IOTF standards. Analyses of the interviews with the children's guardians were used to determine the influence of the family nucleus on obesity.

**Results:**

The children were classified into four groups based on weight percentiles (underweight, normal, overweight and obese). Significant differences between the groups in relation to ethnicity, social and economical status (ρ = 000.0 for all) were found. The following variables were associated with the development of childhood obesity: fathers' obesity (ρ = 0.001), mothers' (ρ = 0.021) and both parents' (ρ = 0.000). There was no significant statistical difference between fathers and mothers who did (ρ = 0.81) or did not work out (ρ = 0.15). Obesity (ρ = 0.07) tended to be less prevalent in the child whose parents were separated. Family history of obesity (OR = 3.3; IC = 2.0 – 5.5; ρ = 0.000) and high social class (OR = 3.0; IC = 1.1 – 7.7; ρ = 0.020) were predictive and independent associated factors.

**Conclusion:**

This study confirms the influence of genetic and/or behavioral factors on the origin of childhood obesity. Thus, effective intervention strategies must be focused not only on the children but on the entire family nucleus.

## Background

Childhood obesity has reached epidemic proportions worldwide, in developed and developing countries [1,2]. The mechanisms responsible for the increasing rates are not completely understood, but excessive weight gain results from the interaction of several factors, including genetic, metabolic, behavioral and environmental influences. The rapid rate of increase in obesity suggests that behavioral and environmental influences play a fundamental role in its development [3,4].

Childhood overweight and obesity are associated with a variety of adverse consequences, such as risk for cardiovascular disease and metabolic disorders. Furthermore, long-term follow-up results indicate that obese children tend to become obese adults [5]. These conditions therefore cause not only widespread effects on health, but also result in a tremendous economic burden [3], thus justifying early intervention, and for this to be more effective, knowledge is required about the characteristics of the population in question.

The aim of this study was to identify family risk factors (socio-economic class, family history of obesity, parents' occupation and marital status) for the development of overweight and obesity in a sample of children from Feira de Santana, BA.

## Methods

Feira de Santana is a northeastern Brazilian city, the second largest in the state of Bahia, has 498.638 inhabitants of whom, 9.2% are children ranging from 5 to 9 years of age. Over 50% of the population, mostly consisting of the mulatto group, has a family income of less than the Brazilian minimum wage per month (U$ 170.00) [6].

The sample comprised 699 children ranging from five to nine years of age (mean age 7.14 ± 1.3 years), at 10 public (n = 415) and 18 private (n = 284) schools. The schools were selected randomly with equal probability for each school and through a three-stage cluster sample technique based on the educational system characteristics, which included 280 schools: 100 public and 180 private. Systematic sampling was used in the third stage. In this way the proportionality between public and private school was obtained and the sample was representative of the whole population, therefore subjects from all socioeconomic classes and ethnicity were included. The local Human Research Ethics Committee reviewed and approved the study. Informed consent and assent was obtained from all guardians and school directors.

Standard calibrated scales and stadiometers were used to determine height (SD), weight (SD), and body mass index (BMI) of all participants. The mean of three measurements was obtained by a group of nursery education students, who were previously trained during school hours. Overweight and obesity were defined as BMI ≥ 85^th ^and the 95^th ^percentiles both for age and gender, respectively, adopting the cut-off obtained from the International Obesity Task Force Study [7]. Under weight and normal weight were also defined using the BMI < 10^th ^and between 10^th ^and 85^th^, respectively [8].

Interviews with the children's guardians were used to evaluate the biological, psychological and social factors studied: fathers' and/or mothers' obesity, parents' professional occupation (work at or outside home), marital status (divorced, married or living together), social and economic class. The highest school education level achieved by either mother or father was used to define social. Parents' education was classified according to school attendance (number of years) into 'low' (< 9 yr), 'middle' (10–12 yr) and 'high" (> 13 yr). The salary earned by either mother or father was used to define economic class and it was stratified into three categories: 'low" was assigned to those whose income per month was ≤ one Brazilian minimum wage (U$ 170.00); 'middle' (from two to ten); and, 'high' (above ten minimum wages). Single parent families were not excluded from the analysis. The maternal and paternal obesity values were included as dichotomous self-reported variables.

Following an initial descriptive analysis, the Chi-square (χ^2^) or Fisher's exact test to test the differences between proportions and a multivariate logistic regression model were used. ρ values < 0.05 were considered statistically significant.

## Results

The sample was classified in two groups based on the school type: 1 (n = 415), from public and 2 (n = 284) from private schools. Baseline characteristics for these groups are shown in Table 1. The prevalence of overweight and obesity were respectively, 6.5% and 2.7% in public schools and 13.4% and 7.0% in private ones (ρ <0.000 for all). Significant differences were found in age (ρ = 0.000), ethnicity (ρ = 0.000), social (ρ = 0.000) and economic class (ρ = 0.000). In public schools, a preponderance of children from the mulatto group and low social and economic class was noted; in the other hand, white children from middle social and economic status predominated in private schools. In spite of this, in both groups the prevalences of parents' excessive weight and outside work activities were similar.

**Table 1:** Baseline demographic characteristics of the sample according to school type

**Variables**		**All**	**Schools**		ρ value
				
		**n = 699**	**Public (n = 415)**	**Private (n = 284)**	
Age (y)		7.1 ± 1.3	7.4 ± 1.2	6.7 ± 1.3	0.000
Gender (boys)		333 (48.0)	188 (45.4)	145 (51.1)	0.142
Ethnic group (mulatto)		306 (43.8)	225 (56.8)	81 (29.0)	0.000
Overweight (%)		64 (9.3)	26 (6.5)	38 (13.4)	0.000
Obesity (%)		31 (4.4)	12 (2.7)	19 (7.0)	0.000
Fathers' occupation (works outside)		603 (86.2)	345 (85.1)	258 (87.7)	0.385
Mothers' occupation (works outside)		365 (52.2)	208 (50.9)	157 (55.3)	0.251
Positive father's obesity history		217 (31.0)	119 (29.5)	98 (35.0)	0.143
	mother's	245 (35.1)	143 (35.1)	102 (36.2)	0.780
	parent's	118 (16.9)	70 (16.9)	48 (16.8)	0.982
Social status	low	424 (60.7)	344 (83.9)	80 (28.3)	0.000
	middle	240 (34.3)	66 (16.1)	174 (61.5)	0.000
	high	29 (4.1)	-	29 (10.2)	0.000
Economic status	low	296 (42.3)	277 (67.4)	19 (6.7)	0.000
	middle	348 (49.8)	130 (31.6)	218 (77.3)	0.000
	high	49 (7.0)	4 (1.0)	45 (16.0)	0.000

Data are means ± SD. n (%). ρ based on Wilcoxon's rank-sum test for continuous variables and χ^2 ^test for categorical variables

In the present study, a high percentage (86%) of agreement between the guardians' perception of children's weights and the clinical evaluation was achieved (Table 2). However, among overweight/obese children, 59% of the guardians (56/95) had a wrong perception of their weight.

**Table 2:** Percentage of agreement between guardians' perception and clinical diagnosis of excessive weight

***Clinical diagnosis***	***Parental information***		Total
		
	**Obese**	**Non obese**	
Obese	39	56	95
Non obese	42	562	604

Total	81	618	699

For better stratification of weight status, the population was also classified into four groups (underweight, normal, overweight and obese) (Table 3). Significant differences with regard to ethnicity (ρ = 0.000), social (ρ = 0.000) and economical status (ρ = 0.000) were found. The prevalence of excessive weight (overweight/obesity) among the three ethnic groups (white, mulatto and black) were 20.2%, 9.6% and 11.1%, respectively (ρ = 0.001). Black children had the lowest social (ρ = 0.000) and economic status (ρ = 0.000) and an association between them and under weight was identified (ρ = 0.000). In addition, overweight and obese children predominate in middle and high social and economic status groups.

**Table 3:** Baseline characteristic of the population stratified according to weight based on weight percentiles

	***Nutritional Status***				
		
	**Under weight (n = 60)**	**Normal weight (n = 544)**	**Over weight (n = 64)**	**Obese (n = 31)**	ρ value
**Ethnic Group**					
white	16 (26.7)	178 (32.7)	36 (56.3)	13 (41.9)	0.001
mulatto	29 (48.3)	269 (49.4)	22 (34.4)	10 (32.3)	0.019
black	15 (25.0)	97 (17.8)	6 (9.4)	8 (25.8)	0.412
**works outside**				
Father's	44 (80.0)	448 (87.2)	50 (79.4)	24 (80.0)	0.439
Mother's	28 (46.7)	258 (48.0)	27 (42.2)	15 (48.4)	0.773
**Positive obesity history**					
Fathers'	18 (30.0)	153 (28.8)	32 (51.6)	14 (45.2)	0.002
Mothers'	18 (30.0)	183 (34.2)	28 (44.4)	16 (51.6)	0.011
Parents'	9 (15.0)	77 (14.2)	20 (31.3)	12 (38.7)	0.000
**Social class**					
low	46 (76.7)	337 (62.5)	28 (44.4)	13 (41.9)	0.000
middle	14 (23.3)	182 (33.8)	29 (46.0)	15 (48.4)	0.002
high	-	20 (3.7)	6 (9.5)	3 (9.7)	0.002
**Economic class**					
low	38 (63.3)	230 (42.7)	19 (30.2)	9 (29.0)	0.000
middle	19 (31.7)	276 (51.2)	36 (57.1)	17 (54.8)	0.017
high	3 (5.0)	33 (6.1)	8 (12.7)	5 (16.1)	0.008

Data are means ± SD for continuous variables and n (%) for categorical. ρ # based on analysis of variance (ANOVA) for continuous variables and ρ for trend for categorical variables

Overweight and obesity were significantly associated with fathers' (ρ < 0.03), mothers' (ρ < 0.03) and parents' (ρ < 0.03) obesity. Obesity tended to be less prevalent in children whose parents were separated (ρ = 0.07) and there was no significant difference between the above-mentioned conditions and the fathers' and mothers' type of occupation (ρ = 0.81; ρ = 0.15). Parents' obesity (OR = 3.5; IC = 1.9 – 6.3; ρ = 0.000) as well as high social class (OR = 3.7; IC = 1.4 – 9.4; ρ = 0.006) were identified as a predictive and independent factors for the development of overweight and obesity.

## Discussion

A human being's weight is determined by the interaction among the genetic inheritance [9-11] and risk factor (RF) recognition is the key to prevention and treatment of weight excess, although these factors are not clearly established in childhood [12]. Reilly et al [9] identified parents' obesity with the background of genetic and environmental mechanisms as a great RF. The main finding of this study was that parents' obesity history and social-economical class play a significant role in the development of childhood overweight/obesity irrespective of ethnicity. When the groups are compared on the basis of weight, excessive weight was significantly associated with parents' positive obesity history, in agreement with the literature [13-15]. Genetic information is sufficient cause for determining obesity, but it is not always necessary, this condition being strongly influenced by the environment children live in, and it is known that the life style adopted by parents is generally transferred to their children, thus perpetuating the overweight phenotype [3].

Childhood obesity has clearly become a serious personal and public health problem for the world population [5,16,17]. Olshansky et al [18] estimated that the current life expectancy at birth in the United States would be 1/3 to 3/4 of a year higher if all overweight adults were to attain their ideal weight and a recent reported described 1.7 billion children as overweight worldwide [19]. A study previously reported from our group showed childhood overweight and obesity prevalence of 9.3% and 4.4% respectively [20]. The prevalence of overweight was 15.2% in a group of Brazilian adolescents [21] and 10.2% at the age of 4 years [22].

Some reports described an inverse relationship between excessive weight and social class [23,24]. In developed countries, the decreasing rates of obesity in the high social class clearly show that the education level may slow down the rise of this condition. In another previous study from our group [5], there was a positive association between overweight and obesity and the presence of television, computer and games (ρ = 000.0). Therefore, especially in developing countries with low-incomes, but with growing rates of food availability, focus on prevention is required through educational programs. In the present study, most of the families from obese children were unaware of this condition and thus had not yet realized the magnitude of the problem and its consequences.

A Brazilian study conducted in the southern region, much richer than northeastern Brazil, showed an increase in the rates of childhood obesity especially in the families with low and middle incomes [26] even though the education level was not analyzed. This pattern is closer to that seen in developed countries. The finding of 8.5% underweight in the present sample, confirmed the presence of a nutrition transition stage in Brazil [27] and this very complex relationship order for an intervention. The majority of parents worked outside home, but no significant association was observed between this variable and the greater prevalence of overweight and obesity.

With regard to parents' marital situation, this study found that children whose parents did not live together tended to have a lower prevalence of overweight/obesity, which could be explained because dietary disturbances caused by adverse psychological conditions do not necessarily imply excessive weight gain [28]. It is also known that when parents separate, there is frequently an important reduction in family income, resulting in decreased access to food.

The results of the present study showed that the family's habits and relations in the genesis of obesity should be appreciated; as they reinforce the importance of family structure and emphasize the parents' influence on children, not only from a genetic, but also from a behavioral point of view. Being alert to this new focus may modify the intervention strategy, and direct it not only toward the child, but allow it to include the whole family.

## Conclusion

To sum up, the data of the present study confirm the multifactorial character of childhood overweight and obesity and show the importance of biological, psychological and social factors in its development and emphasize the role of families as a target for prevention and treatment. In this population, the parents' obesity was highlighted as an independent predictive risk factor for development of weight excess.

## Competing interests

The author(s) declare that they have no competing interests.

## Authors' contributions

AMO conceived the idea and developed the drafted the manuscript. ACO, MSA and LA participated on data collection. All authors participated in writing the paper.

## Pre-publication history

The pre-publication history for this paper can be accessed here:


